# The University of Maryland, Dental Department

**Published:** 1886-09

**Authors:** 


### Editorial, Etc.

---

THE UNIVERSITY OF MARYLAND will begin the session of 1886-87, in its Dental Department with a larger class than has ever before in its history been present on the first day of the regular sessions. This institution, from the ability displayed by its graduates in different parts of the world has acquired a reputation of which there is reason to be justly proud, and the increased number of each class at the annual sessions is an evidence of the appreciation of the efforts made by its Faculty to maintain a dental school of the highest character and to thoroughly educate students for the successful practice of dentistry in all its branches.

Although extensive additions have been recently made for the accommodation of the steadily increasing number of students, all of whom are afforded equal facilities, the question already presents itself for more space.

That the success of this Department of Dentistry is beyond any example heretofore known is beyond question, and the prospects for the future are of the most promising character. Students fiom many foreign countries, some represented by a large number, and the majority of the States of this country have already matriculated for the session of 1886-87, the exercises of which begin on the first day of October, 1886.

